# A qualitative study on the mismatch between health literacy and nursing needs in the postoperative recovery of breast cancer: nurse and survivor perspectives

**DOI:** 10.1186/s12912-025-04264-6

**Published:** 2026-01-08

**Authors:** Yue Ma, Lei Hou, Xiaofang Fan, Xiaoxia Xu

**Affiliations:** 1 Operation Department, The Affiliated Cancer Hospital of Zhengzhou University & Henan Cancer Hospital, No. 127, Dongming Road, Jinshui District, Zhengzhou, Henan China; 2 Breast Surgery Ward, The Affiliated Cancer Hospital of Zhengzhou University & Henan Cancer Hospital, Zhengzhou, Henan China; 3 Nursing Department, The Affiliated Cancer Hospital of Zhengzhou University & Henan Cancer Hospital, No. 127, Dongming Road, Jinshui District, Zhengzhou, Henan China

**Keywords:** Cancer, Nursing, Needs, Health literacy, Qualitative study

## Abstract

**Objective:**

This study aimed to explore the mismatch between health literacy and care needs at various stages from the perspectives of both breast cancer survivors (BCS) and nurses.

**Methods:**

This descriptive study utilized purposive sampling to conduct semi-structured in-depth interviews with postoperative BCS and nurses at a hospital in Henan, China, from April to August 2025. Data processing was executed using NVivo 12 software, and content analysis was employed for data analysis.

**Findings:**

A total of 25 BCS and 9 nurses participated in the study. Four themes and sixteen sub-themes were identified from the analysis: (1) discharge transition period: construction of safety nets and perceived gaps, (2) treatment and rehabilitation period: the “compliance” dilemma and the absence of empowerment, (3) navigating the transitional teachable moment: the support vacuum from clinical outcomes to life’s new beginnings, (4) the cross-cutting nexus: communication and interaction.

**Conclusion:**

Discrepancies exist between BCS and nurses regarding health literacy perceptions and care needs across different survivorship phases. Addressing these gaps requires establishing a new model that integrates dynamic health literacy assessment, relational communication, collaborative rehabilitation planning, and systematic support.

## Introduction

Breast cancer continues to pose a significant global health challenge and remains the leading cause of cancer-related deaths among women worldwide. According to estimates from GLOBOCAN, over 2.3 million new cases of breast cancer are diagnosed annually (as of 2022), accounting for nearly a quarter of all female cancer cases [1]. Thanks to advancements in older detection and treatment, survival rates following a breast cancer diagnosis have improved markedly [2]. Many BCS are expected to live for more than five years after surgery [3]. However, for these BCS, the completion of primary treatment does not signify the end of their journey; rather, it marks the beginning of a complex and prolonged survival phase that extends for many years [4].

The survival trajectory of BCS is not monolithic; rather, it consists of distinct phases, each presenting unique challenges. These phases are typically defined as the transition period (from active treatment to survivorship), early survivorship, and long-term survivorship, each characterized by evolving physical, psychological, and social sequelae [5]. Common challenges include managing treatment-related side effects (such as lymphedema, fatigue, and pain) [6], overcoming fear of recurrence [7], coping with body image disturbances and sexual dysfunction [8], and addressing return-to-work issues along with financial toxicity [9]. The nature and intensity of these challenges continuously evolve throughout the survivorship journey, necessitating adaptive coping strategies and ongoing support. Health literacy is the ability to access, understand, appraise, and apply health information, and effect longterm health outcomes [10, 11]. Adequate health literacy enables BCS to effectively communicate with healthcare providers, comprehend complex follow-up care instructions, make informed decisions regarding their health, and engage in essential shared decision-making and self-management behaviors, thereby allowing them to choose healthy lifestyles that directly impact their quality of life and potentially reduce unnecessary healthcare expense expenditures [12].

In addition to the crucial role of personal health literacy in maintaining long-term high-quality survival, the care needs that survivors must address cannot be overlooked [13]. These needs include, but were not limited to, postoperative symptom management; informational support regarding long-term effects, rehabilitation, and monitoring; psychological support for anxiety and depression; and practical assistance for social reintegration [14]. During the long-term survival phase, BCS might require nursing support to manage the late effects of treatment, prevent recurrence, and transition back to normal life. Researches indicated that nurses played an indispensable and multifaceted role in addressing these evolving needs throughout the survival continuum [15]– [16]. They typically served as the primary point of contact for BCS, providing education, emotional support, and care coordination [17]. Their ongoing close contact with BCS enabled them to effectively assess health literacy levels, identify unmet needs, deliver tailored education, and facilitate timely referrals [18].

The effectiveness of nursing interventions inherently depended on a profound and meticulous understanding of patients’ life experiences, health literacy, and perceived needs at each stage [18]. The level of health literacy among BCS influenced their perception, expression, and prioritization of personal care needs [19]. Conversely, nurses assessed these needs through their clinical perspective, which was shaped by their understanding of the BCS’ health literacy. A notable discrepancy occurred when there was a disconnect between the lived experiences and self-perceived needs of BCS — affected by their health literacy—and the clinical assessments made by nurses, which were influenced by their perceptions of the BCS’ health literacy [20]. While previous studies had mapped the quality of life, common symptoms, and supportive care needs of BCS [10, 13, 15, 21], and quantitative researches had identified prevalent needs linked to various demographic and clinical variables [22, 23], a notable gap remains. However, it was critically important to note that few studies had explicitly compared and contrasted the perspectives of nurses and BCS (the dual caregiver-patient perspective) regarding health literacy and care needs across different survival stages. This gap was critical because a mismatch between patient-reported and nurse-perceived needs, compounded by disparities in health literacy, may lead to misaligned care priorities, ineffective communication, and suboptimal recovery outcomes [24]. The postoperative period for BCS represented a vulnerable and evolving phase, as they transition from intensive hospital care to self-management at home [25]. Responsibilities such as managing complex symptoms, understanding follow-up plans, and recognizing warning signs created an urgent need for comprehensible information [26]. Providing adequate informational support during this critical time was essential. It equipped survivors with the necessary tools to navigate the early challenges of recovery, alleviate anxiety, and establish effective self-management practices that extend throughout their survivorship journey [27].

Comprehensively understanding the perspectives of both BCS and nurses was essential for developing effective interventions that enhanced health literacy and addressed the care needs [28]. By comparing the viewpoints of BCS and nurses, we identified communication gaps, discrepancies in understanding, and opportunitied to improve the quality of care [29]. Qualitative research could provide a rich description of patients’ experiences, offering profound insights into the emotional and social dimensions of survivorship [30]. Therefore, this qualitative study aimed to explore and contrast the perceptions of both BCS and nurses regarding the influence of health literacy on postoperative care needs across the early and long-term survival trajectory.

## Methods

### Design

This descriptive qualitative study was conducted from April 2025 to August 2025. Semi-structured interviews were employed to facilitate the interview process. The interview was featured by a semi-structural style in line with the consolidated criteria for reporting qualitative research (COREQ) guideline [31].

### Participants

Participants were recruited from the Affiliated Cancer Hospital of Zhengzhou University & Henan Cancer Hospital. It is a nationally recognized center with extensive experience in breast cancer treatment, located in the capital of central China. Purposive sampling was employed for participant selection to ensure that the chosen individuals could provide in-depth information relevant to the research questions. The inclusion criteria for BCS in this study were: (a) a pathological diagnosis of breast cancer, (b) post-surgical status with a stable condition, (c) awareness of their own medical condition, and (d) normal communication and expression abilities. Exclusion criteria included: (a) the presence of severe organic diseases; (b) individuals with mental, cognitive, or visual impairments that could hinder cooperation in this study; and (c) unwillingness to participate for any reason. Registered nurses with over two years of experience in the care of BCS were selected as interviewees, as they were deemed to possess substantial relevant expertise. Furthermore, all participating nurses were interviewed based on the principles of informed consent and voluntary participation. A maximum variation sampling strategy was adopted for participant selection. To this end, this approach enriched the data spectrum, thereby allowing us to effectively capture key thematic meanings and identify the factors underlying the nurse-patient disparity in perceived care needs. Participant screening resulted in 40 eligible BCS and 15 eligible nurses. Of the BCS, 9 declined to participate due to ongoing treatment needs, lack of interest, or an unwillingness to undergo audio recording. Among the nurses, 3 refused participation, all citing work-related constraints.

In this study, we classified BCS at various stages based on the Timing It Right framework [32] and their clinical characteristics. The core concept of TIR framework was that patients’ care needs and information requirements dynamically evolved alongside the progression of their illness and the passage of time. Therefore, the support provided to patients must ‘deliver the right content at the right time.’ This framework divided the prolonged recovery process into continuous phases, each characterized by distinct core tasks and challenges [33]. It transcended static assessments of health literacy by exploring how patients perceive, understand, and utilize health information during each specific critical transition period, as well as how their information needs and cognitive abilities evolve over time. Through this framework, qualitative research could uncover cognitive mismatches between nurses and patients regarding ‘what information was needed at what time,’ thereby elucidating why uniform health information support failed and establishing a theoretical foundation for developing precise interventions that provided ‘appropriate information’ at the ‘right time.’ In this study, the post-operative period for BCS was categorized into three phases: a) the period from post-operative stabilization to discharge, b) the home treatment and rehabilitation phase (before the first follow-up examination), and c) navigating the transitional teachable moment, (Following the first follow-up examination). To prevent overlap among participants across different stages, the study categorized the participants into three distinct groups: A, B, and C. The sample size for each group was determined based on data saturation, which was defined as the point at which no new themes emerged from the thematic analysis, and further data collection failed to yield additional insights into the participants’ experiences [30]. For instance, in Group B, the 8-bit BCS did not reveal any new information following the interviews. To validate the saturation of information, two additional interviews were conducted; however, no new themes were identified, resulting in a total of 10 completed interviews.

### Data collection

The interview guide encompassed demographic information, comprehensive details regarding disease and treatment, and a structured question outline aligned with the research objectives. Two clinical nursing experts evaluated the content of the interview guide, and two pre-interviews (not included in the study results) were conducted to ensure its rigor. The final version of the interview guide comprised the following sections: (1) Common questions for both BCS and nurse: e.g., what does “health literacy” mean to people in the context of recovering from breast cancer surgery?, (2) For BCS interview: e.g., can you describe a time after your surgery when you felt confident about managing your care at home (e.g., wound care, symptom management)?, (3) For nurse interview: e.g., what are the key components of the nursing support and education you provide to breast cancer patients after surgery?. (Table 1)

**Table 1:** The interview guide

**Section 1: Common Questions for Both BCS and Nurse**
1. In your own words, what does “health literacy” mean to people in the context of recovering from breast cancer surgery?
2. What do you think are the most important skills or abilities for a patient to have in order to manage their recovery effectively?
3. How do you think good health literacy affects a patient’s recovery journey? Conversely, what challenges arise when there are difficulties with health literacy?
**Sect. 2: BCS Interview Guide**
A. Daily Manifestations of Health Literacy & Personal Experiences:
1. Can you describe a time after your surgery when you felt confident about managing your care at home (e.g., wound care, symptom management)? What helped you feel confident?
2. Can you describe a time when you felt confused, unsure, or anxious about what to do during your recovery? What was the situation and how did you handle it?
3. When you need to explain a new symptom or a concern to your nurse, how do you usually describe it? Is it easy or difficult?
B. Content of Services Received & Evaluation of Nursing Support:
4. What kind of information, support, or coaching from the nurses has been most helpful to you since your surgery? Please give an example.
5. Was there any information or support you needed but felt you didn’t receive, or that could have been provided in a better way? Please explain.
6 How would you describe the role of a nurse in your recovery? What makes a “good” nurse in your experience?
C. Expectations for the Future:
7. Looking back, if you could give one piece of advice to healthcare providers on how to better support patients like you, what would it be?
**Sect. 3: Nurse Interview Guide**
A. Content of Services Provided & Self-Evaluations:
1. What are the key components of the nursing support and education you provide to breast cancer patients after surgery? (Probe: discharge planning, symptom management, psychosocial support)
2. What part of your patient education and support do you feel is most effective? Why?
3. Are there areas of patient support that you find particularly challenging or feel less confident in providing? (e.g., addressing body image issues, sexual health, financial concerns)
B. Perceptions of Patient Performance & Challenges Faced:
4. In your experience, what are the most common challenges or barriers that prevent patients from following the recommended care plans after surgery? (Probe: knowledge, skills, motivation, external factors)
5. How do you assess whether a patient truly understands the health information you’ve provided and is prepared to manage their care at home?
6. What are the biggest challenges you face in your daily practice when trying to provide optimal support to these patients? (Probe: time constraints, resource limitations, communication barriers)
C. Future Expectations:
7. If you had more resources or support, what is one thing you would change or improve in the way patients are supported through their recovery journey

The interviews were conducted in a one-on-one format, consisting of a single interviewer and a participant. The interview setting was mandated to be private and quiet to ensure confidentiality. Participants selected the interview times based on their convenience. The interviewer possessed extensive experience in qualitative research methodologies. Prior to the interviews, the department nurse assisted the interviewer in clarifying the purpose, content, and intent of the interviews to the participants. All interviews were recorded, and the interviewer meticulously documented the participants’ body language and emotional expressions, such as laughter, tears, and frowns, to gain a deeper understanding of their genuine thoughts. To alleviate the burden of hospital travel for BCS in Group B, researchers conducted video interviews using the WeChat platform. In contrast, participants in Groups A and C, who were receiving treatment in the hospital, underwent face-to-face interviews on-site. Aside from the mode of interview (online versus offline), the procedures and content of the interviews remained consistent across all groups. Each BCS was assigned a unique alphanumeric identifier (e.g., A1, B1, C1), and the numeric identifier for nurses was Nx. The duration of each interview ranged from 30 to 40 min. Following informed consent, all participants, whether conducted online or in-person, were audio-recorded in their entirety. Demographic and disease-related information of the participants was obtained from medical records to avoid disrupting the narrative flow.

### Data analysis

The qualitative content analysis method developed by Graneheim and Lundman was employed for the data analysis in this study [34]. This methodology facilitated a systematic description and interpretation of the data, providing a structured approach to examining the contexts, meanings, and interrelations of participants’ care needs. The analysis process was conducted in several stages: (1) data preparation, (2) organization of linguistic units (words, phrases, sentences, or paragraphs), (3) unit classification through coding, (4) assessment of coding consistency, and (5) extraction of key thematic results aligned with the TIR Framework. Content analysis was carried out independently by two researchers. Nvivo 12 software was utilized for the data analysis.

### Rigour

To ensure the rigor and credibility of the interview data, this study employed triangulation and member checking strategies. Triangulation involved conducting interviews with nurses, whose responses were cross-verified through an analysis of hospital work protocols and documentation. Member checking targeted the BCS, where participants were invited to review the transcribed texts and preliminary thematic findings for validation. Their feedback was incorporated to refine the research outcomes. Additionally, to guarantee reproducibility, all procedures—including interview guides, data transcription, and analysis steps—were meticulously documented. Regular team discussions and reflective practices, such as recording potential biases, were conducted to enhance the trustworthiness and reliability of the findings.

### Ethics

The study was approved by the Ethics Committee of The Affiliated Cancer Hospital of Zhengzhou University & Henan Cancer Hospital (No.2023 − 363) and adhered to the principles outlined in the Declaration of Helsinki. All participants were assured of the following rights: (a) the right to withdraw from the study at any time; (b) participant information will be utilized solely for the purposes of this study; (c) refusal to participate in an interview will not result in any form of discrimination; (d) potential benefits of participation, including health education and health consultation.

## Results

A total of 25 participants were interviewed. The BCS’ ages ranged from 38 to 54 years, with disease durations spanning from two months to six months, as shown in Table 2. A total of 9 nurses had 11 ~ 25 years of experience and were directly involved in the care of breast cancer patients. (Table 3) Four themes and sixteen sub-themes were identified. (Table 4)

**Table 2:** Demographic information of BCS

No.	Age (years)	Educational stage	Marriage status	Postoperative Period (month)	Occupational status
A1	38	college	married	2	work
A2	38	middle school	married	5	work
A3	40	middle school	married	2	work
A4	42	college	married	5	work
A5	50	middle school	married	5	no
A6	43	college	married	5	work
A7	44	middle school	married	5	work
A8	45	college	married	5	no
B1	42	middle school	married	5	work
B2	50	secondary vocational school	married	5	no
B3	44	postgraduate	married	5	work
B4	45	college	married	5	no
B5	42	college	married	5	no
B6	50	middle school	married	5	no
B7	48	middle school	married	4	work
B8	41	college	married	4	no
B9	45	middle school	married	4	work
B10	49	primary school	married	4	no
C1	49	middle school	married	4	no
C2	48	secondary vocational school	married	4	work
C3	50	middle school	married	4	no
C4	54	secondary vocational school	married	5	no
C5	52	middle school	married	5	work
C6	54	primary school	married	5	no
C7	51	middle school	married	5	no

**Table 3:** Demographic information of nurses

*N*(x)	Gender	Age(years)	Nurse level	Education level	Years of experience in nursing
N1	female	35	N3	bachelor’s degree	12
N2	female	44	N3	bachelor’s degree	20
N3	female	38	N3	bachelor’s degree	18
N4	female	37	N3	bachelor’s degree	16
N5	female	36	N2	bachelor’s degree	14
N6	female	42	N3	master degree	18
N7	female	33	N2	bachelor’s degree	11
N8	female	43	N3	bachelor’s degree	25
N9	female	39	N3	bachelor’s degree	18

**Table 4:** Themes and Sub-Themes identified in the analysis

Theme	Sub-theme
Theme 1: Discharge Transition Period: Construction of Safety Nets and Perceived Gaps	Sub-topic 1.1: Active Engagement in Treatment and Adherence to Decisions
	Sub-topic 1.2: Discontinuity in Information Delivery and Comprehension
	Sub-theme 1.3: Technical Security and Emotional Reassurance
	Sub-theme 1.4: Rational Assessment and Anxiety-Induced Responses
Theme 2: Treatment and Rehabilitation Period: The “Compliance” Dilemma and the Absence of Empowerment	Sub-theme 2.1: The Gap Between Knowledge and Action
	Sub-theme 2.2: Attribution of Defects and Contextual Disorders
	Sub-theme 2.3: Quantitative Language Use and Narrative Expression
	Sub-theme 2.4: Resource Allocation and Proactive Engagement
	Sub-theme 2.5: Body Image Concerns and Sexual Health Issues
Theme 3: Navigating the Transitional Teachable Moment: The Support Vacuum from Clinical Outcomes to Life’s New Beginnings	Sub-theme 3.1: Understanding How to “**r**ebuild Life”
	Sub-theme 3.2: Termination of Relationships and Ongoing Dependency Dynamics
	Sub-theme 3.3: Illness Impacting Life Focus Shifts
	Sub-theme 3.4: General Recommendations Versus Tailored Plans for Individuals
Theme 4: The Cross-Cutting Nexus: Communication and Interaction	Sub-theme 4.1: Time Efficiency Versus Depth of Relationships
	Sub-theme 4.2: Evaluative Questions Versus Empowering Inquiries
	Sub-theme 4.3: Collaborative Efforts and Continuity in Relationships

### Theme 1: Discharge transition period: construction of safety Nets and perceived gaps

This period captured the critical transition from hospital to home, highlighting a fundamental disconnect in how nurses and BCS perceive and achieve a sense of safety.

#### Sub-Topic 1.1: Active engagement in treatment and adherence to decisions

Both nurses and BCS shared the perception that a critical dimension of health literacy during this phase was the BCS’ active exploration of treatment options and their high level of adherence to the agreed-upon decisions.

*They’re very cooperative with the post-surgery treatment and seem genuinely interested in learning more about it. N1*

*I’m very concerned about my recovery. I look up information online every day to learn more. A2*

#### Sub-Topic 1.2: Discontinuity in information delivery and comprehension

Nurses perceived information delivery as a singular task, whereas BCS, grappling with postoperative vulnerabilities, encounter challenges in absorbing all the information simultaneously. This necessitated repeated communication, thereby creating a gap between the transmission of information and actual comprehension.

*Typically*,* we conduct comprehensive missionary work followed by the distribution of various materials. N8*

*The information is indeed contained within the materials and videos we have provided*,* but*,* they (patients) exhibit reluctance to engage with them. N9*

*The missionary work occurred during a period of my weakness*,* which significantly hindered my ability to retain information. A4*

#### Sub-Theme 1.3: Technical security and emotional reassurance

Safety for nurses was rooted in the objective monitoring of clinical parameters (e.g., drain output, incision sites), while BCS equated safety with the availability of ongoing emotional support and the reassurance of accessible professional backup.

*The priority is teaching them to identify signs of infection or lymphedema. Accurate reporting is key to preventing complications. N4*

*What I needed most was to know someone (nurse) was there for me. A call response that I could hear in time*,* just to hear ‘You’re going to be okay’. A6*

#### Sub-Theme 1.4: Rational assessment and anxiety-induced responses

Nurses expressed an expectation for BCS to rationally triage concerns based on clinical urgency, in contrast to BCS who described help-seeking behaviors driven by a pervasive fear of recurrence and a need for immediate reassurance for any anomalous sensation.

*We encourage them to use their resources wisely. Calling about every minor ache reduces our capacity for genuine emergencies. N2*

*Every twinge of pain sent me into a panic. I needed to call not just for an answer*,* but for the peace of mind that it wasn’t the cancer coming back. A2*

### Theme 2: Treatment and rehabilitation period: the “Compliance” dilemma and the absence of empowerment

The focus of this period was on evaluating the effectiveness of home-based rehabilitation. Nurses perceived the inadequate implementation and outcomes of rehabilitation plans as issues of BCS ‘compliance,’ whereas BCS contended that they required ‘empowerment’ rather than judgment.

#### Sub-Theme 2.1: The gap between knowledge and action

The core challenge for BCS health literacy at this stage lied not in the acquisition of knowledge, but in its translation into sustainable health practices.

*We don’t know exactly what they’re doing at home*,* but judging by the results*,* they probably aren’t following through with the exercises. N4*

*I know some recommendations are essential*,* but I always struggle to stick to them. There are both personal and practical reasons for it. B3*

#### Sub-Theme 2.2: Attribution of defects and contextual disorders

Nurses attributed lapses in adherence to rehabilitation plans to BCS deficits in motivation or comprehension, while BCS consistently highlighted insurmountable contextual barriers, such as debilitating fatigue, pain, and family responsibilities.

*The home-based rehab exercises we assign to patients have been tested repeatedly and are well within their ability. If they’re not completed*,* it’s usually a matter of willpower or comprehension. N5*

*After I got home from chemotherapy*,* I felt extremely weak. The amount of rehab exercises was a real challenge for me. B4*

*Some of the exercises require special equipment*,* but I’m not too keen on paying for it. I’m hoping to find some cheaper alternatives. B7*

#### Sub-Theme 2.3: Quantitative language use and narrative expression

A communicative chasm existed between nurses’ need for quantifiable, objective symptom data to guide clinical decisions and BCS’ natural expression of their symptoms through subjective, narrative descriptions of their lived experience.

*It’s valuable to hear about patients’ home rehab experiences*,* but if the descriptions are too detailed*,* it becomes difficult to sift through and extract the key information I need. N1*

*I need them (patient) to rate their pain on a scale of 1 to 10. However*,* they often can’t give a specific number. They usually just say*,* ‘I feel terrible*,*’ which isn’t enough for me to adjust their treatment plan effectively. N9*

*How can I possibly describe this feeling with a number? This is a deep*,* constant pain that affects my whole body. The numbers just don’t capture it. B8*

#### Sub-Theme 2.4: Resource allocation and proactive engagement

Nurses perceived their role as informing BCS about available psychosocial resources, considering their duty fulfilled upon provision. BCS, however, described profound barriers to accessing these resources and expressed a need for nurses to proactively and sensitively engage them in conversations about psychological distress.

*We have pamphlets for support groups and counseling. The information is there if they want it. N7*.

*Community nurses should play a larger role. I certainly recognize that we need to provide them with the necessary support to make that happen. N6*.

*I was too depressed to even think about calling a counselor. I wished my nurse would have sat down and said*,* ‘It’s normal to feel this way*,* let’s talk about it. B10*.

#### Sub-Theme 2.5: Body image concerns and sexual health issues

Both parties tended to avoid discussions related to body image and sexual health. Nurses, constrained by concerns or perceived role boundaries, typically wait for BCS to initiate these conversations. Meanwhile, BCS, burdened by feelings of shame and embarrassment.

*Unless the patient brings it up*,* we don’t typically discuss intimacy. It’s a private matter. N4*

*I felt so unattractive and broken. But how do you ask your nurse about that?. B2*

*I’m really struggling with whether or not to bring this up with the nurse. I feel too embarrassed; it’s kind of shameful. B8*

### Theme 3: Navigating the transitional teachable moment: the support vacuum from clinical outcomes to life’s new beginnings

BCS experienced a sense of abandonment during periodic follow-up visits, as the clinical focus on disease surveillance diverged from their need to reconstruct their lives beyond cancer.

#### Sub-Theme 3.1: Understanding how to “rebuild Life”

BCS faced a fundamental challenge regarding health literacy, a paradigm shift from a passive compliance model focused on “treating diseases” under the guidance of medical teams to an active self-management model centered on “rebuilding lives” led by individuals.

*As patients’ physical condition improves*,* a growing emphasis is placed on their quality of life. N*

*Individuals who effectively manage their illness while also cultivating a fulfilling life are considered exemplary models. N2*

*Health is defined by the capacity to self-assess one’s physical state daily and to create living conditions that support well-being. C3*

#### Sub-Theme 3.2: Termination of relationships and ongoing dependency dynamics

From the nursing perspective, the conclusion of active treatment signaled an appropriate endpoint for the intensive nurse-patient relationship, with a transition to routine surveillance. BCS, conversely, described this transition as a precipitous drop into a “support vacuum,” yearning for continued guidance.

*Our active role concludes with treatment. They are transitioned back to their primary care physician for follow-up. N2*

*When treatment stopped*,* the support stopped. I felt cast adrift*,* alone with my fears about the future. C6*

#### Sub-Theme 3.3: Illness impacting life focus shifts

Nursing communications during follow-up were predominantly focused on monitoring for recurrence. BCS’ primary concerns, however, had shifted to managing long-term sequelae (e.g., cognitive changes, fatigue) and navigating the challenges of reintegrating into social and professional roles.

*Our follow-up calls focus on checking for new symptoms*,* like lumps or pain*,* that could indicate recurrence. N6*

*The nurse asked if I’d found a new lump. I wanted to talk about how I couldn’t concentrate at work anymore*,* but it didn’t seem like the right time. C2*

#### Sub-Theme 3.4: General recommendations versus tailored plans for individuals

Nurses tended to offer generic health promotion advice, while BCS expressed a critical need for personalized, practical strategies tailored to their specific energy levels, lifestyle, and long-term goals.

*We advise a healthy diet and regular physical activity—standard recommendations for all survivors. N8*

*It’s true that not every patient receives an individualized plan. However*,* for special cases—such as cancer patients with co-existing conditions*,* or for those who specifically request it—we do provide additional guidance from a nursing perspective. N9*

*‘Eat healthy and exercise’ is not a plan. I needed a realistic guide. ‘What kind of exercise can I do with this fatigue? How can I manage my diet when I’m too tired to cook?‘ C7*

### Theme 4: The Cross-Cutting nexus: communication and interaction

Maintaining effective communication and interaction between nurses and BCS was a fundamental aspect that permeates the entire treatment process. This dynamic not only reshaped the nursing experience but also influenced BCS’ expectations regarding care.

#### Sub-Theme 4.1: Time efficiency versus depth of relationships

Driven by systemic pressures, nurses prioritized task-efficient, transactional communication. BCS, in contrast, deeply valued the relational connection built through moments of unstructured, empathetic conversation, which made them feel seen as individuals.

*Our workload demands that we focus on the essential clinical tasks during each encounter. N1*

*The nurses who took a moment to ask about my family*,* not just my wound*,* made me feel like I mattered. That connection was as healing as the medicine. B5*

#### Sub-Theme 4.2: Evaluative questions versus empowering inquiries

Nurses’ questions, intended to assess knowledge or adherence, were frequently perceived by BCS as tests, triggering defensiveness. In contrast, collaborative, open-ended questions made BCS feel supported and empowered as partners in their care.

*Even some basic terminology can confuse patients. We can’t rule out that their educational background might make it hard for them to understand these terms. N4*

*I’m sometimes puzzled by the questions they suddenly ask. I don’t always see where they’re coming from. A3*

*It’s necessary to have a clear explanation for everything. But obviously*,* this patient education task is sometimes difficult for junior nurses*,* yet they are often the ones doing it. C7*

#### Sub-Theme 4.3: Collaborative efforts and continuity in relationships

Nursing practice emphasized the importance of teamwork, which included collaboration among nurses, as well as between medical staffs and other healthcare professionals. BCS appreciated the trust and efficiency that stem from the continuous relationships established with their designated nurses.

*Frequent patients often develop a preference for a specific nurse. However*,* that nurse isn’t always available*,* as we work as a team. We cannot adjust our entire team’s schedule to accommodate one patient’s request. N2*

*I would prefer to be hospitalized when both my doctor and my preferred nurse are present. I feel more reassured when I can talk to them. A7*

## Discussion

This study, by investigating the health literacy and nursing needs of nurses and BCS following surgery, revealed biases in the understanding of health literacy and nursing needs throughout the entire postoperative survivor trajectory. The research identified safety and emotional needs prior to patient discharge, empowerment needs during the rehabilitation period, and the establishment of self-management strategies for life reconstruction in the long-term survival phase. Both nurses and BCS recognized the significant value of effective communication and interaction in addressing nursing needs at various stages, and the shift in focus of these needs also highlighted the necessity of establishing effective liaison mechanisms between nurses and patients. Our findings emphasized the importance of rectifying discrepancies in patient-nurse communication to promptly address care needs. Furthermore, this study advocate for the establishment of a proactive support system characterized by active follow-up, comprehensive companionship, and genuine empowerment to assist BCS in restarting their lives. This contrasts sharply with the conventional ‘one-time’ care plan, whose effectiveness is often confined to the hospital setting.

The findings of this study indicated that both nurses and BCS acknowledged the highly positive treatment compliance and health management behaviors of BCS from the postoperative period to the early discharge phase, consistent with the research by Khaliq et al. [35]. However, differences in interpreting health literacy and defining safety during this stage led to discrepancies between nursing services and care needs. Nurses primarily conceptualize health literacy as the BCS’ ability to understand, adhere to, and accurately report clinical protocols, focusing on objective clinical parameters as safety indicators. In contrast, patients demonstrated proactive health maintenance by actively seeking informational support and building strong nursing partnerships [36]. However, due to the difficulty BCS face in effectively receiving health education information all at once, especially in their vulnerable post-surgery state, a gap arises between information delivery and functional understanding. This gap was not merely a failure in communication but reflected a conflict between the clinical management paradigm and the survival paradigm. Furthermore, BCS equated safety not only with clinical stability but also with continuous emotional support and immediate reassurance to alleviate pervasive fears of relapse [37]. This divergence explained why nurses might perceive frequent requests for assistance as non-urgent, while BCS consider them crucial for psychological survival. To bridge these gaps, transitional care should evolve from a transactional information delivery model to a relational, patient-centered guidance model [38]. This evolution involved implementing structured, repeated education after discharge, integrating emotional support into clinical protocols, and providing tailored, accessible resources. This comprehensive approach effectively addressed survivors’ informational and emotional needs, thereby creating a truly effective safety net [39].

This study identified a fundamental paradigm conflict between nurses and BCS during treatment and rehabilitation, specifically the transformation from knowledge acquisition to the conversion of that knowledge into sustainable practices [40]. Central to this conflict were the concepts of “compliance” and “empowerment.” Nurses primarily attributed rehabilitation outcomes to BCS noncompliance, perceiving it as a deficiency in motivation or understanding [41]. In contrast, patients explicitly expressed a need for empowerment, pointing to significant environmental barriers—such as debilitating fatigue, pain, and family responsibilities—that were often overlooked. This indicated that the challenges encountered in home-based rehabilitation did not solely arise from BCS’ lack of knowledge. The failure to bridge the gap between acquiring health information and its implementation within the complex realities of BCS’ lives would continue to impede the effectiveness of home-based rehabilitation [42]. Moreover, this study found that communication discrepancies exacerbate the dilemma of “obedience versus authorization.” Nurses operated within a clinical framework that prioritized quantitative, objective data to guide decision-making. In contrast, BCS expressed their experiences through subjective, narrative descriptions of suffering. This divergence in focus not only hindered effective symptom management but also contributed to BCS feeling neglected. Furthermore, certain topics, such as body image and sexual health, were collectively avoided, leaving some of the BCS’ needs unaddressed [43]. Addressing these challenges necessitated that healthcare professionals proactively established partnerships with BCS, facilitating their transition from mere knowledge acquisition to self-management skills. This process involved several key components: First, rehabilitation plans should be collaboratively developed with patients, recognizing and addressing their unique environmental barriers, rather than being unilaterally prescribed [44]. Second, communication training must enable nurses to better comprehend BCS’ narratives and employ patient-centered communication techniques to gather clinically relevant information [38]. Third, psychosocial support should be actively integrated into routine follow-ups; nurses need to be trained and empowered to sensitively and proactively initiate discussions regarding psychological distress, body image, and sexual health, thereby normalizing these issues as essential components of rehabilitation [45].

This study found that the primary objective of long-term survival was for BCS to attain a high quality of life while coexisting with cancer through the process of “rebuilding life,” which was characterized by the establishment of BCS’ self-health management capabilities. The findings of Zhao et al. supported this conclusion [46]. However, this study identified a disconnect, or even a rupture, between the continuity of care provided by nurses and the lived experiences of BCS. From a nursing perspective, the cessation of active treatment upon patient discharged appropriately signifies the conclusion of intensive therapeutic relationships and a transition towards monitoring for recurrence and promoting health [47]. Nevertheless, BCS perceived this transition as abruptly entering a “support vacuum,” feeling abandoned at a critical time when they require guidance to navigate the new normal of survival. This gap was further exacerbated by the misalignment of priorities: care communication during follow-up primarily focuses on detecting physical signs of recurrence, while BCS’ main concerns had shifted to managing debilitating long-term sequelae (e.g., cancer-related fatigue, cognitive changes) and reintegrating into social and occupational roles [48]. Consequently, providing general health advice, such as “eat healthily” and “exercise,” was perceived by BCS as insufficient and dismissive. This approach failed to meet their needs for personalized and practical strategies that were tailored to their individual energy levels, lifestyles, and long-term goals. To bridge this gap, care should evolve from mere monitoring to encompass structured interventions that proactively manage long-term effects, promote psychological adjustment, and provide tailored guidance for life reintegration. Such an evolution would empower BCS to become architects of their own sustainable well-being [49].

This study found that the alignment of care services with care needs during the transition from hospital to home fundamentally hinged on the quality of nurse-patient communication and interaction. The quality of communication significantly shaped the therapeutic alliance and was a key determinant of BCS’ mental health and their perceived experience of care throughout the survival process [50]. Nurses faced a dilemma, and they prioritized time efficiency and task orientation, fulfilling clinical responsibilities primarily through communication forms such as assessments and education [51]. This led to a reliance on closed-ended questions to assess compliance and an emphasis on team-based care models, which inevitably compromised the continuity of the nurse-patient relationship. However, from the patient’s perspective, this approach served as a barrier to establishing a therapeutic connection. BCS expressed a long-term need for empathy and relational communication, which was essential for relational continuity and empathetic dialogue [52]. This need remained constant and underpinned their specific information requirements at discharge, their desire for empowerment during rehabilitation, and their longing for guidance throughout long-term survival.

### Implications

The traditional training model, which focuses on knowledge transmission and procedural compliance, is inadequate to meet the evolving needs of patients. Therefore, the training of breast cancer nurses must undergo a fundamental paradigm shift from being ‘educators’ to becoming ‘enablers’. Specifically, this shift entails: (1) Dynamic health literacy assessments that enable nurses to identify the varying needs of patients at different stages of rehabilitation; (2) The development of relational communication skills, allowing nurses to foster therapeutic partnerships through techniques such as narrative medicine, motivational interviewing, and shared decision-making [41, 53, 54]; (3) Collaborative rehabilitation planning, which trains nurses and patients to co-develop individualized rehabilitation plans that consider environmental factors, thereby facilitating the translation of knowledge into practice; (4) Systematic support capabilities that equip nurses with structured intervention methods for effective follow-up, management of long-term effects, and integration of psychosocial support [55]. The ultimate goal of this model is to reconceptualize health literacy as a dynamic construct and to genuinely empower patients to become the primary agents of their own health. Furthermore, at the policy level, it is imperative for policymakers to promote the establishment of an integrated care pathway that encompasses the entire survival period, ensuring continuity and a seamless transition from hospital to community and family support [56]. At the research level, future efforts should focus on collaboratively designing and rigorously evaluating initiatives such as contact support programs or shared care plans led by medical professionals [57]. Longitudinal studies should be conducted to verify their effectiveness in bridging paradigm differences, enhancing the consistency of health literacy, and improving the long-term quality of life for patients. This approach aims to construct a new paradigm for the rehabilitation of breast cancer patients, centered on life reconstruction.

### Limitation

To the best of our knowledge, limited qualitative research has been conducted to explore the health literacy and care needs of BCS across various postoperative stages in China, particularly studies that integrate perspectives from both nurses and patients. Our findings offered valuable references for strategies designed to enhance health literacy and nursing services for BCS, ultimately improving their quality of life. However, this study had certain limitations. Firstly, despite the researchers’ expertise in qualitative research, the findings remained susceptible to subjective interpretation, which introduced a degree of bias. Secondly, given the particular psychological vulnerability of BCS preoperatively, this phase was excluded from our investigation. This exclusion limits our understanding of the patients’ complete psychological trajectory throughout their illness. Thirdly, several BCD were interviewed via video. Compared to offline interviews, online interviews may lead to the loss or distortion of non-verbal information, such as gestures and body language that cannot be fully observed, as well as facial expressions that may be distorted due to network signals. It could potentially affect the accuracy of the research results.

Fourth, all participants were recruited from the same tertiary hospital, which possesses specific healthcare resources and cultural characteristics that may limit the direct transferability of the findings to other institutions. Additionally, participants’ sensitivity towards audio recording, which led some patients to decline participation, may have somewhat affected the depth of credibility. Finally, although most BCS tended to return home or to their communities for long-term rehabilitation, the study did not include interviews with community nurses, primary caregivers, and other relevant groups, which might limit the findings.

## Conclusion

This study identifies a significant misalignment between the nursing care model and the lived experiences of BCS. This misalignment subsequently leads to cognitive disparities in health literacy and leaves patients’ care needs unmet throughout their survivorship trajectories. To bridge this gap, the rehabilitation training system for breast cancer specialist nurses should undergo a fundamental restructuring. Specifically, it should transition from the traditional “knowledge transmitter” model to a novel “patient enabler” model. This new model should integrate dynamic health literacy assessment, relational communication, collaborative rehabilitation planning, and systematic support. Future efforts should prioritize the development of integrated care pathways that ensure continuity of support, as well as rigorously evaluate the impact of such patient-centered interventions on long-term quality of life.

## Data Availability

To uphold ethical standards and protect participant privacy, the data collected from this study will remain confidential and will not be disclosed unless explicitly mandated by the research protocol or regulatory authorities.

## Abbreviations

BCS: Breast cancer survivor
COREQ: Consolidated criteria for reporting qualitative research
